# Earliest Archaeological Evidence of Persistent Hominin Carnivory

**DOI:** 10.1371/journal.pone.0062174

**Published:** 2013-04-25

**Authors:** Joseph V. Ferraro, Thomas W. Plummer, Briana L. Pobiner, James S. Oliver, Laura C. Bishop, David R. Braun, Peter W. Ditchfield, John W. Seaman, Katie M. Binetti, John W. Seaman, Fritz Hertel, Richard Potts

**Affiliations:** 1 Department of Anthropology and Institute of Archaeology, Baylor University, Waco, Texas, United States of America; 2 Department of Anthropology, Queens College & NYCEP, Flushing, New York, United States of America; 3 Human Origins Program, National Museum of Natural History, Smithsonian Institution, Washington, D. C., United States of America; 4 Department of Anthropology, Illinois State Museum, Springfield, Illinois, United States of America; 5 Research Centre in Evolutionary Anthropology and Palaeoecology, School of Natural Sciences and Psychology, Liverpool John Moores University, Liverpool, United Kingdom; 6 Department of Anthropology, George Washington University, Washington, D. C., United States of America; 7 Research Laboratory for Archaeology and the History of Art, University of Oxford, Oxford, United Kingdom; 8 Department of Statistical Science, Baylor University, Waco, Texas, United States of America; 9 Department of Biology, California State University, Northridge, California, United States of America; 10 Palaeontology Section, Earth Sciences Department, National Museums of Kenya, Nairobi, Kenya; University of Oxford, United Kingdom

## Abstract

The emergence of lithic technology by ∼2.6 million years ago (Ma) is often interpreted as a correlate of increasingly recurrent hominin acquisition and consumption of animal remains. Associated faunal evidence, however, is poorly preserved prior to ∼1.8 Ma, limiting our understanding of early archaeological (Oldowan) hominin carnivory. Here, we detail three large well-preserved zooarchaeological assemblages from Kanjera South, Kenya. The assemblages date to ∼2.0 Ma, pre-dating all previously published archaeofaunas of appreciable size. At Kanjera, there is clear evidence that Oldowan hominins acquired and processed numerous, relatively complete, small ungulate carcasses. Moreover, they had at least occasional access to the fleshed remains of larger, wildebeest-sized animals. The overall record of hominin activities is consistent through the stratified sequence – spanning hundreds to thousands of years – and provides the earliest archaeological evidence of sustained hominin involvement with fleshed animal remains (i.e., persistent carnivory), a foraging adaptation central to many models of hominin evolution.

## Introduction

Unique among extant primates, modern humans are anatomically adapted to regularly consume substantial amounts of vertebrate animal tissues (meat, organs, etc.). Over the last several million years, the hominin gastrointestinal tract has evolved from a chimpanzee-like large-intestine-dominated configuration well adapted for digesting fruits and other plant parts (as well as the occasional small mammal) to a more carnivore-like small-intestine-dominated form well suited for extracting complex nutrients from animal remains [1], [2]. Increased consumption of animal tissues likely fueled brain expansion in the genus *Homo* [1], [3]–[5], and may have helped to facilitate initial hominin dispersals out of Africa (ca. 1.8 Ma) [3], [6]–[7].

Despite its central role in many models of hominin evolution [1]–[8], however, relatively little is known about the timing and nature of the emergence of persistent hominin carnivory. From an archaeological perspective, the appearance of flaked lithic technology around ∼2.6 Ma is often though to reflect increased levels of hominin interest in, and involvement with, animal remains [8]–[12]. Associated faunal evidence, though, is largely unknown prior to 1.8 Ma, limiting opportunities to test these and related hypotheses using the zooarchaeological record.

In addition, past efforts to integrate archaeologically-derived inferences of Oldowan hominin diet with broader issues of theory have often been hindered by a range of analytical and interpretive challenges. Setting aside the overall rarity of assemblages for a moment, three of these constraints are particularly noteworthy. First, the earliest archaeofaunal assemblages are generally associated with small analytical datasets; with most collections having relatively few specimens, poor bone surface preservation, or both [10]–[15]. The earliest sites (ca. 3.4–2.3 Ma), those from Ethiopia (Dikika, Gona, Bouri, and Hadar), and Kenya (Lokalalei), for instance, each preserve evidence of one or more hominin butchery acts within analytically-small faunal collections [10–15, but see 16]. These records demonstrate that Oldowan hominins acquired and consumed animal tissues, at least on occasion. Yet given the limited amount of behavioral information potentially recorded in *any* small assemblage, it is uncertain if these records reflect initial hominin forays into carnivory – i.e., the occasional meal – or something substantively more typical and adaptively important [17], [18].

Second, the earliest assemblages are, by-and-large, isolated points in time and space. This is potentially problematic on two fronts. When considering the record on a site-by-site basis, each of these assemblages formed in less than 10^1–3^ years [10]–[15], [19]. Foraging activities documented at this time-scale, though, while certainly reflecting past hominin behaviors, need not reflect evolved patterns of behavior (i.e. adaptations). As a result, we are often left with open questions of ‘forays’ versus ‘fundamental shifts in foraging activities’ when trying to interpret the record. Moreover, these assemblages are rarely recovered in clear stratigraphic succession [10]–[15]. As a result, demonstrating any continuity in hominin behavior through time – especially relative to a single ecological context (the appropriate frame of reference) – is often fraught with considerable analytical and interpretive difficulties.

Lastly, behavioral inferences derived from the archaeofaunal record can often be equivocal, even in cases where there is an abundance of well-preserved remains. Numerous studies of FLK 22 *Zinj* (1.84 Ma; Olduvai Gorge, Tanzania), for instance, have returned diverse often mutually-exclusive interpretations of Oldowan hominin diet and behavior [20]–[42]. This disjunction is potentially attributable to conceptual and methodological differences among analysts [43], but may also reflect the difficult and often subjective task of teasing apart the behavioral roles and material contributions (if any) of both hominins and carnivores in site formation activities [39], [43], [44].

Given these limitations, how might researchers differentiate between ‘initial forays into the carnivorous realm’ [2], [3], [11], [13] and ‘persistent carnivory’ when studying the earliest archaeofaunal record? To do so requires a stratified series of relatively large assemblages, each with clear evidence of sustained and abundant hominin involvement with fleshed animal remains, with each assemblage sampling a relatively discrete period of time (i.e., sampling behavior at an ecological-scale), and with the sum of assemblages providing an unambiguous record of sustained carnivorous behavior persisting at geological/evolutionary time scales (i.e., at 10^3+^ years). At present, the earliest archaeological evidence of this adaptation is found either in the stratified assemblages of Bed I, Olduvai Gorge, Tanzania (1.86–1.75 Ma) [21,25,33,35; though see 20], or, somewhat more conservatively, in the Okote Member, Koobi Fora, Kenya (∼1.5 Ma) [44].The relatively recent dates of these assemblages, though, pose a challenge for models of hominin paleobiology that posit an earlier appearance for this adaptation – particularly so for models that associate increased carnivory with the emergence and early evolutionary history of the genus *Homo* up to 1 million years earlier [e.g., 1–3,5–7; though see 46–48]. Recent zooarchaeological discoveries at Kanjera South, Kenya provide new data relevant to this debate. Here we report on three large, well-preserved, stratified archaeofaunal assemblages that date to ∼2.0 Ma and collectively provide the earliest material evidence of persistent hominin carnivory.

## Materials and Methods

**Figure 1:**
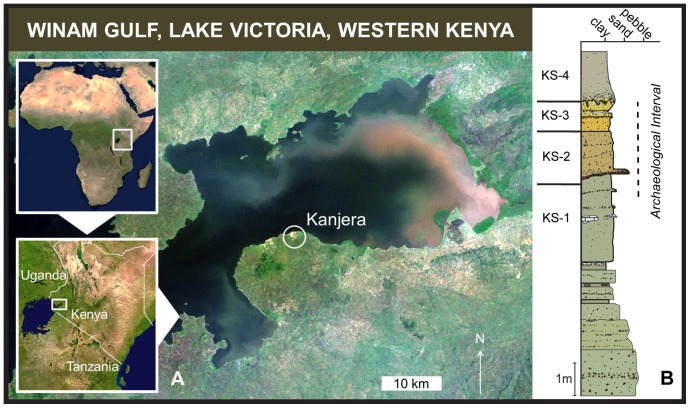
Location of Kanjera along the modern shoreline of Lake Victoria, East Africa. (A) Kanjera lies to the immediate northeast of Homa Mountain, a volcanic complex active from the middle Miocene to the Pleistocene. The Winam Gulf fills the western end of the Nyanza Rift, an E-W graben with origins in the early Miocene. (B) Beds KS-1 through KS-3 of the Kanjera Formation (Southern Member) sample floodplain and low-aspect channel contexts originally deposited between the mountain and the nearby shores of a shallow lake [49]. Satellite imagery from USGS and NASA.

Kanjera South (KJS) is located on the southern shores of the Winam Gulf of Lake Victoria, southwestern Kenya (0°20′24″S, 34°32′16″E) (Figure 1). A relatively small (∼0.5 km^2^) amphitheater of stratified fluvial-lacustrine sediments, this locality has yielded *in situ* archaeological materials from each of its three lowermost beds (KS-1 though KS-3) [49]–[54]. The presence of the proboscidean *Deinotherium* sp. and the suid *Metridiochoerus andrewsi* provides a minimum age of 1.7 Ma for the sediments; and the equid *Equus* provides a maximum age of 2.3 Ma. The presence of the Olduvai subchron (1.77–1.95 Ma) in the uppermost bed, KS-5, further constrains the archaeological levels to ∼2.0 Ma [50].

Three excavations set along a ∼50 m transect of outcrop have recovered several thousand well-preserved, spatially-associated, faunal and lithic artifact specimens (Table 1) [49]–[55]. When limiting ourselves to the archaeological interval (total depth ∼3.1 m), Excavation 1 (169 m^2^) samples beds KS-1 through KS-3; Excavation 2 (15 m^2^) and Excavation 5 (4 m^2^) sample KS-3. Clear lithostratigraphic correlations among excavations, short distances between excavations, and an absence of purposeful spatial organization of materials within beds or excavations allows faunal materials from KS-3 to be collectively considered a single assemblage [17].

**Table 1:** Faunal and lithic inventory.

Bed	Total NISP	Macro-mammal NISP	Macro-mam. MNI	Principal fauna (% NISP, % MNI)	Lithics
KS-1	982	975 (525)	25	Bovid (92.4, 72.0), Equid (4.4, 8.0), Suid (1.5, 8.0), Hippo (0.2, 4.0)	179
KS-2	2190	2153 (886)	40	Bovid (82.6, 67.5), Equid (11.6, 10.0), Suid (0.9, 5.0), Hippo(1.0, 2.5)	2533
KS-3	491	470 (172)	16	Bovid (77.9, 68.8), Equid (4.7, 6.3), Suid (0.6, 6.3), Hippo (14.0,12.5)	171

NISP (number of identified specimens) and MNI (minimum number of individuals) are defined and quantified following the literature [43]. ‘Total NISP’ reflects the sum of specimens recovered with coordinate data and included in this study. Tens of thousands of non-identifiable bone and tooth fragments <2 cm are omitted from this study. Fossils from conglomeratic facies (CP levels) are poorly preserved [49], and are likewise excluded from this study: KS-2CP (n = 259), KS-3CP (n = 102). Macro-mammals are defined here as weighing >5 kg. Macro-mammal NISP values are total sums and, in parentheses, the sum of specimens identified beyond Linnean class. %NISP and %MNI include macro-mammals only. Faunal and lithic counts are from the literature [17], [55].

Fossils and artifacts were recovered *in situ* by experienced excavators using lightweight hammers and awls [49]–[51]. Sediments were excavated in 1 m×1 m squares and 5 cm levels, with levels following natural stratigraphic units whenever possible. Recovered materials were individually numbered, piece-plotted using a Topcon total station, lifted, and individually bagged. Excavated sediments were further sieved through 1 mm mesh, resulting in the recovery of additional specimens.

Paleoenvironmental analyses indicate that the assemblages formed on a grassy plain set between a freshwater lake and the wooded slopes of nearby hills and mountains. The recovered faunas consist primarily of grassland-adapted bovids (*Parmularius, Antidorcas*), equids (*Equus*), and suids (*Metridiochoerus*), with water-dependent taxa (e.g., *Hippopotamus*, *Crocodylus*, and reduncine bovids) also present in limited numbers. Isotopic analyses of dental enamel and pedogenic carbonates concordantly indicate a grassland setting at KJS [49]–[52].

Site formation studies indicate that the assemblages were neither formed nor significantly winnowed by water flow [17], [49]. Fossils and artifacts are outsized clasts relative to the surrounding clays, silts, and fine-to-medium sands; and the overall abundance and taxonomic diversity of faunal remains far surpass natural landscape accumulation norms [49], [50]. When coupled with the results of previous taphonomic analyses [17], [49], biological agents of site formation are indicated for each of the assemblages. The vertical distribution of materials, deposit depths, and estimated rates of sedimentation and pedogenesis suggest that faunal and artifactual materials accumulated relatively rapidly over a period of decades to hundreds of years per bed. As the stratified assemblages formed both rapidly and recurrently [50], the record from KJS provides a rare opportunity to explore ancient foraging behaviors with regard to both shorter-term ecological and longer-term evolutionary dynamics.

We report here on the zooarchaeological record of bovid remains. These dominate the assemblages in terms of overall abundances (representing a minimum of 56 individuals), and are amenable to analysis using published protocols and experimental datasets [21]–[30], [56]–[63]. Analytically, we group remains by bed (e.g., ‘KS-1’, ‘KS-3’) rather than by excavation [49]. We further sort specimens by body size class [21], grouping animals into ‘small’ (e.g., Grant’s gazelle, *Gazella granti*) and ‘medium’ (e.g., Topi, *Damaliscus lunatus*) sizes. Extinct bovids of intermediate size and weight (e.g., *Parmularius sp*.) are treated as medium-size animals. Larger bovids (e.g., buffalo, *Syncerus caffer*) are poorly represented in the assemblages and are not treated in detail here. Following convention, we incorporate taxonomically-unidentifiable long bone fragments in all appropriate analyses.

In our study of bone surface modifications, three investigators (JVF, BLP, and JSO) jointly analyzed specimens, shared observations, and discussed interpretations before providing individual assessments of bone damage [17]. Analysts employed low–power magnification (10×-40×) and strong light sources to identify modifications. They attributed agency (e.g., hominin, carnivore) to modifications only after excluding all possible alternatives (including potential confounds detailed in [32], [64]–[69]).

Values for minimum numbers of skeletal elements (MNE) reflect considerations of animal size and developmental age, extensive refitting efforts, and, for long bones, element identification of shaft portions [21]. High-survival elements (HSE) include the cranium, mandible, humerus, radius, metacarpal, femur, tibia, and metatarsal [61]. Point estimates of Shannon evenness follow published methods [30], [70], whereas interval estimates are constructed using Bayesian models [71].

## Results and Discussion

Bone surface modification frequencies are known to accurately reflect the timing and context of both hominin and carnivore involvement with animal remains [22–25,56–58; though also see discussions in 16,26]. We use them here to assess the identity and sequence of actors and behaviors responsible for forming and modifying the assemblages.

**Figure 2:**
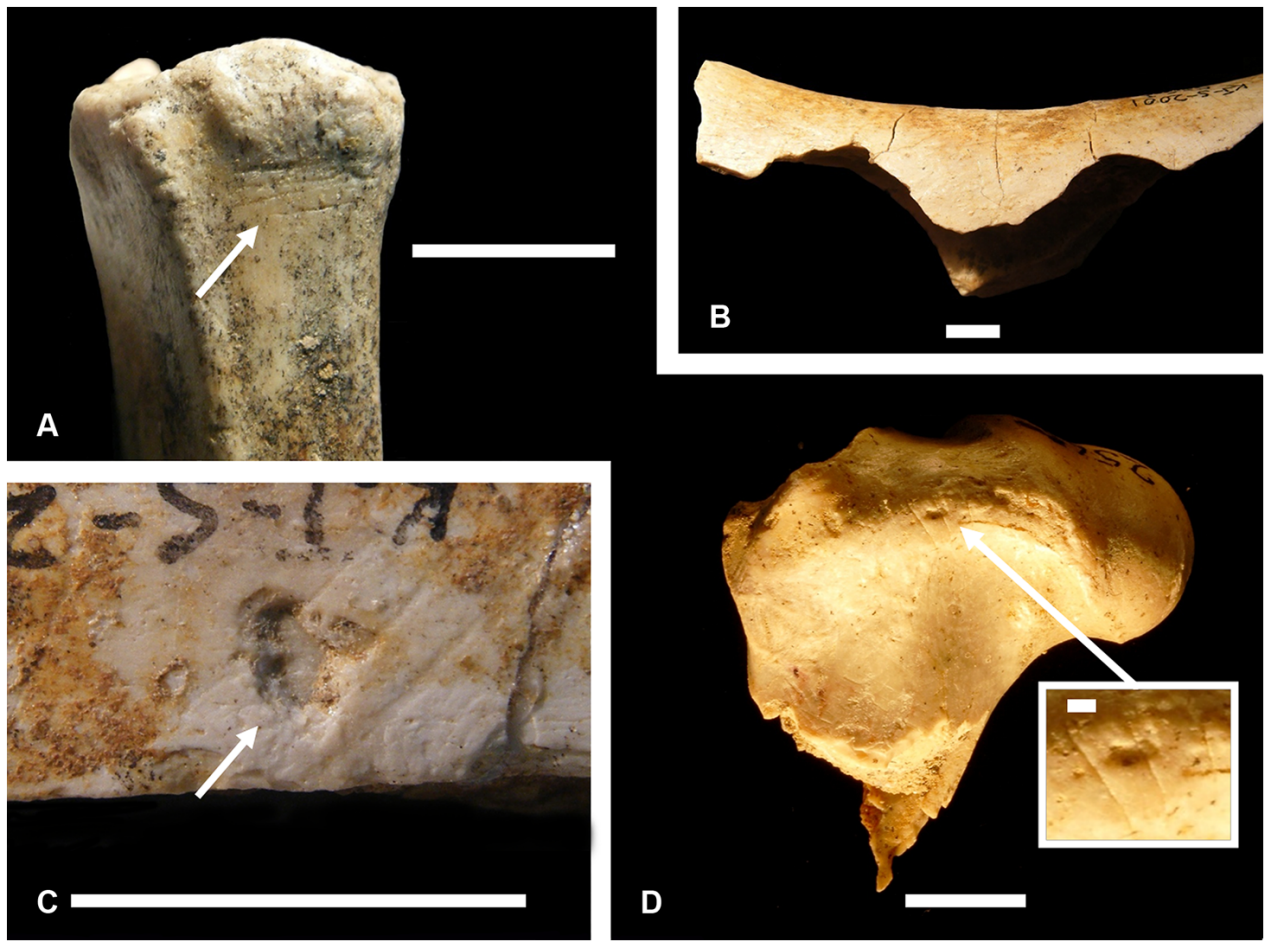
Bone surface modifications. (A) KJS 7472, a small bovid metatarsal from KS-2 bearing cut marks; (B) KJS 7379, a medium-sized bovid humerus from KS3 bearing pair of hammerstone notches, the specimen is also cut-marked (not figured); (C) KJS 5447, a mammal limb bone shaft fragment from KS-2 with percussion pit and striae, the specimen is also cut-marked (not figured); (D) KJS 2565, a small bovid femur from KS-2 with numerous cut marks. Scale is 1 cm in panels (A-D); 1 mm in the panel (D) close-up. Specimen numbers are field designations, not KNM accession numbers.

Hominin-modified specimens (i.e., fossil bones bearing cut marks and/or hammerstone percussion damage) are present through the entire KS-1 through KS-3 sequence (Table 2 and Table S1). These specimens provide unambiguous evidence of hominin processing of bovid remains (Figure 2), and indicate a functional relationship between artifactual and faunal materials. When considering the anatomical placement of cut marks, we report bone damage consistent with both defleshing and disarticulation activities [17]. Frequencies of cut-marked limb specimens range from 1.9% to 6.3% in summed (i.e., total bed) assemblages, with similar frequencies observed irrespective of analyst, bed, or animal body size. The overall uniformity of these results suggests a relatively consistent pattern of carcass exploitation through time (within-analyst test for the homogeneity of cut mark frequencies across beds: homogeneity not falsified, all p-values >0.1).

**Table 2:** Bone surface modification frequencies.

	Epiphyseal Fragments (EPI)				Mid-Shaft Fragments (MSH)			
Bed	TM %	CM %	PM %	N	TM %	CM %	PM %	N
KS-1	18, 24, 18	3, 3, 3	0, 0, 0	34	8, 10, 8	2, 4, 4	9, 9, 10	96
KS-2	13, 17, 11	6, 8, 11	3, 8, 8	64	9, 16, 12	1, 3, 3	4, 5, 8	207
KS-3	9, 9, 0	0, 0, 0	0, 0, 0	11	5, 11, 2	2, 0, 0	5, 2, 14	44
Sum	14, 18, 12	5, 6, 7	2, 5, 5	109	8, 14, 10	1, 3, 3	6, 6, 9	347

Modifications detailed by long bone portion [22]–[24], bed, and analyst. Epiphyseal fragments (EPI) bear at least some of the proximal or distal articular surface. Mid-shaft fragments (MSH) are diaphyseal specimens that lack cancellous bone on medullary surfaces. Bone modifications follow the literature [17 and references therein], and include tooth marks (TM: pits, scores, furrows), cut marks (CM), and percussion marks (PM: pits, striae). Bone modification frequencies are listed by analyst: Ferraro, Pobiner, and Oliver (in order from left to right). Samples are bovid and taxonomically-indeterminate long bone specimens (i.e., humerus, radius, metacarpal, femur, tibia, metatarsal, or ‘long bone shaft fragment’), ≥2 cm in length, from body sizes 1–3 (i.e., small and medium-sized) [21], with ‘very good’ to ‘excellent’ bone surface preservation (i.e., surface conditions 4–5 [17]) and without recent or geological fractures. Data for summed body sizes, including ‘size indet’.

In addition, numerous cut-marked rib specimens reflect recurrent hominin defleshing of axial skeletons of both small and medium-sized animals. In KS-2, 9.7% to 12.9% of smaller-bodied rib specimens (N = 31) and 5.0% to 7.5% of medium-bodied rib specimens (N = 40) bear cut marks (with ranges reflecting multiple analysts’ interpretations). This evidence clearly indicates the repeated tool-mediated removal of soft tissues. Numerous hammerstone-percussed specimens in each assemblage also indicate repeated hominin exploitation of within-bone food resources (Table 2 and Figure 2). From a comparative perspective, frequencies of hominin-induced bone surface modifications are consistent with values recorded from a number of similarly analyzed Early Pleistocene anthropogenic faunal assemblages from East Africa (e.g., BK, Olduvai Gorge; Table S2) [72]–[74].

**Figure 3:**
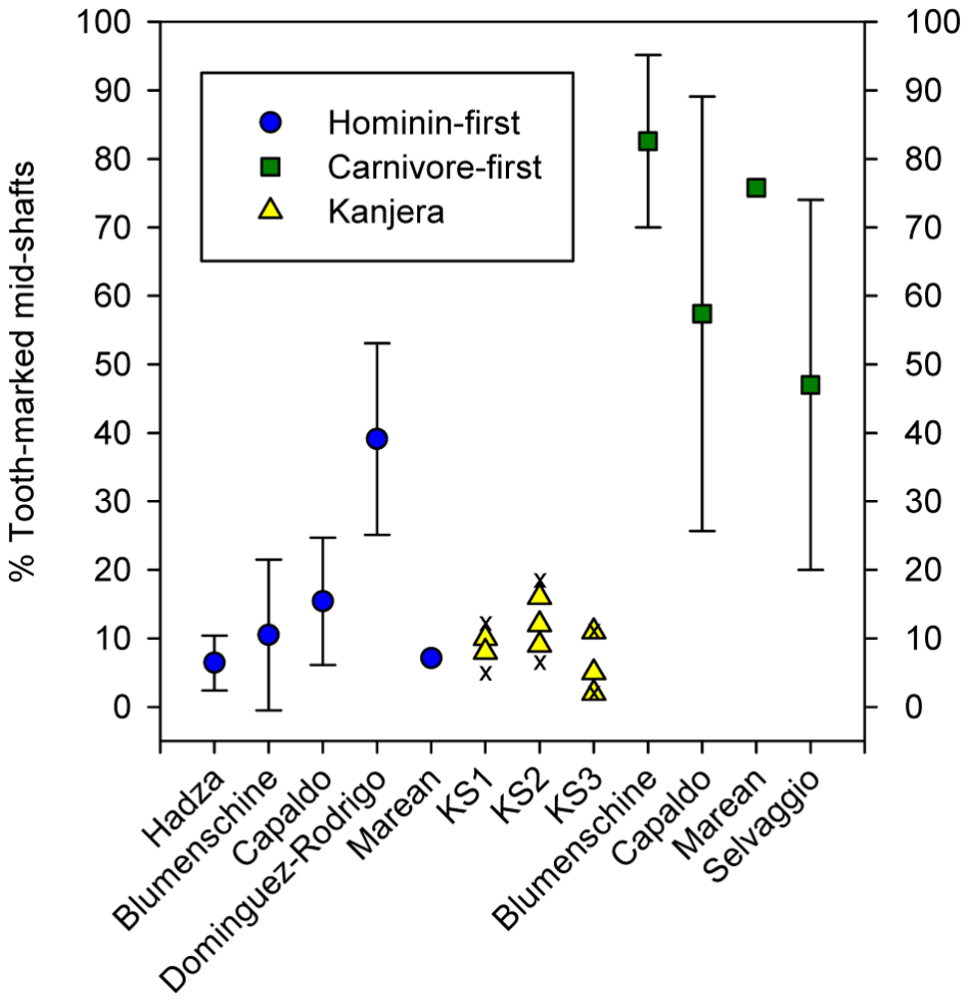
Tooth-marked mid-shaft fragments: results from experimental assemblages and excavations at KJS. Figure follows a published model [26]. Hominin-first assemblages refer to remains initially defleshed and demarrowed by hominins, then subsequently exposed to large-bodied carnivores (primarily hyenas). Carnivore-first assemblages refer to remains initially defleshed and/or demarrowed by large-bodied carnivores (primarily hyenas and/or lions). Data for body sizes 1–4 [21]. Modern data (with single standard deviations where available) derived from the literature [23]–[26], [56]–[58]. KJS frequencies are from Table 2 and Table S1. Multiple symbols for KJS indicate the results of multiple analysts. X’s indicate minimum and maximum estimates of damage (see Table S1).

**Figure 4:**
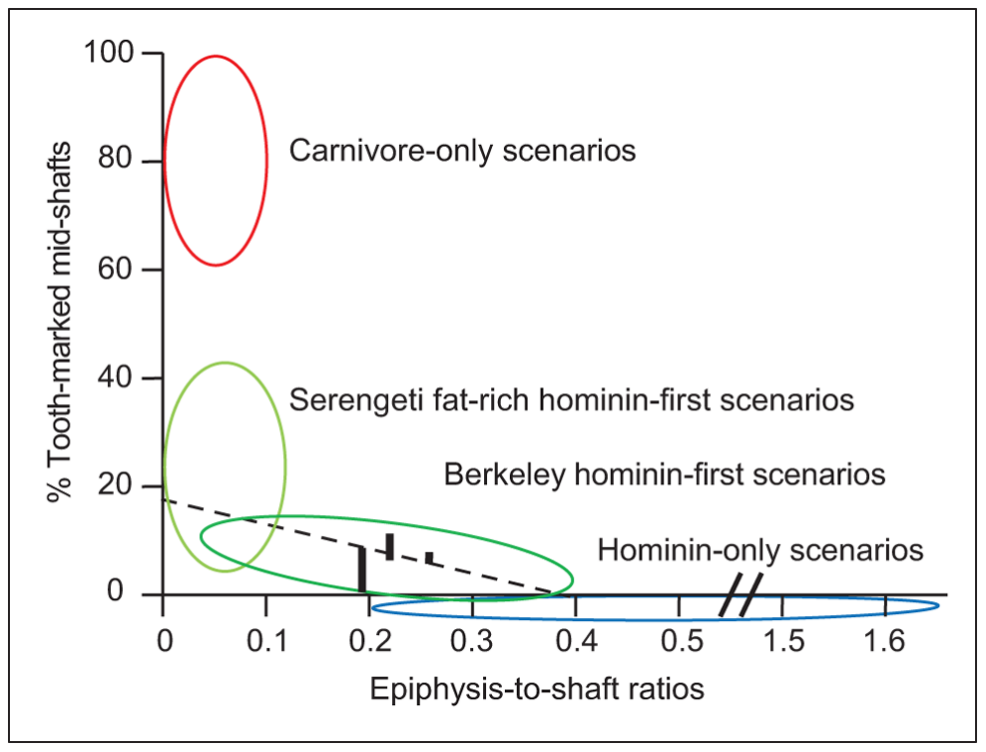
Tooth-mark frequencies and long bone portion representation: results from modern experiments and excavations at KJS. Portions defined in Table 2 and Table S1. ‘Shafts’ include both near-epiphyseal and mid-shaft specimens. Complete bones are not included in epiphysis-to-shaft calculations (number of complete bones = 2, 7, and 1; beds KS-1 through KS-3, respectively). Ellipses outline the range of results in experimental feeding scenarios involving: carnivores-only, hominins-only, or a sequence of hominins-then-carnivores (i.e., ‘hominin-first’). The dashed line is a published least-squares regression for hominin-first scenarios [22]. Hominin-only scenarios have no tooth marks, hence the placement of the ellipse beneath the x-axis. KJS data from Table 2 are for summed body sizes. KJS epiphysis-to-shaft ratios: 0.26, 0.22, and 0.19 for beds KS-1 though KS-3 respectively. KJS tooth-mark data displayed as solid vertical bars, with bars representing the range of analysts’ results. Results from Kanjera are consistent with hominin-first contexts.

Carnivore-damaged specimens (e.g., tooth-marked remains) reflect the actions of additional agents of assemblage modification. Looking at summed (i.e., total bed) assemblages of long bones, <25% of epiphyseal specimens and <17% of midshaft fragments bear tooth-marks (Table 2). Similar frequencies are observed irrespective of bed or animal body size, reflecting some regularity in carnivore feeding behaviors through time (within-analyst homogeneity not falsified for most pairs, all p-values >0.04). Relative to data derived from reference assemblages, tooth-mark frequencies at KJS are indistinguishable from scenarios in which carnivores had secondary access to previously defleshed and demarrowed bone refuse [23]–[26], [56]–[58] (Figure 3, Figure 4).

**Figure 5:**
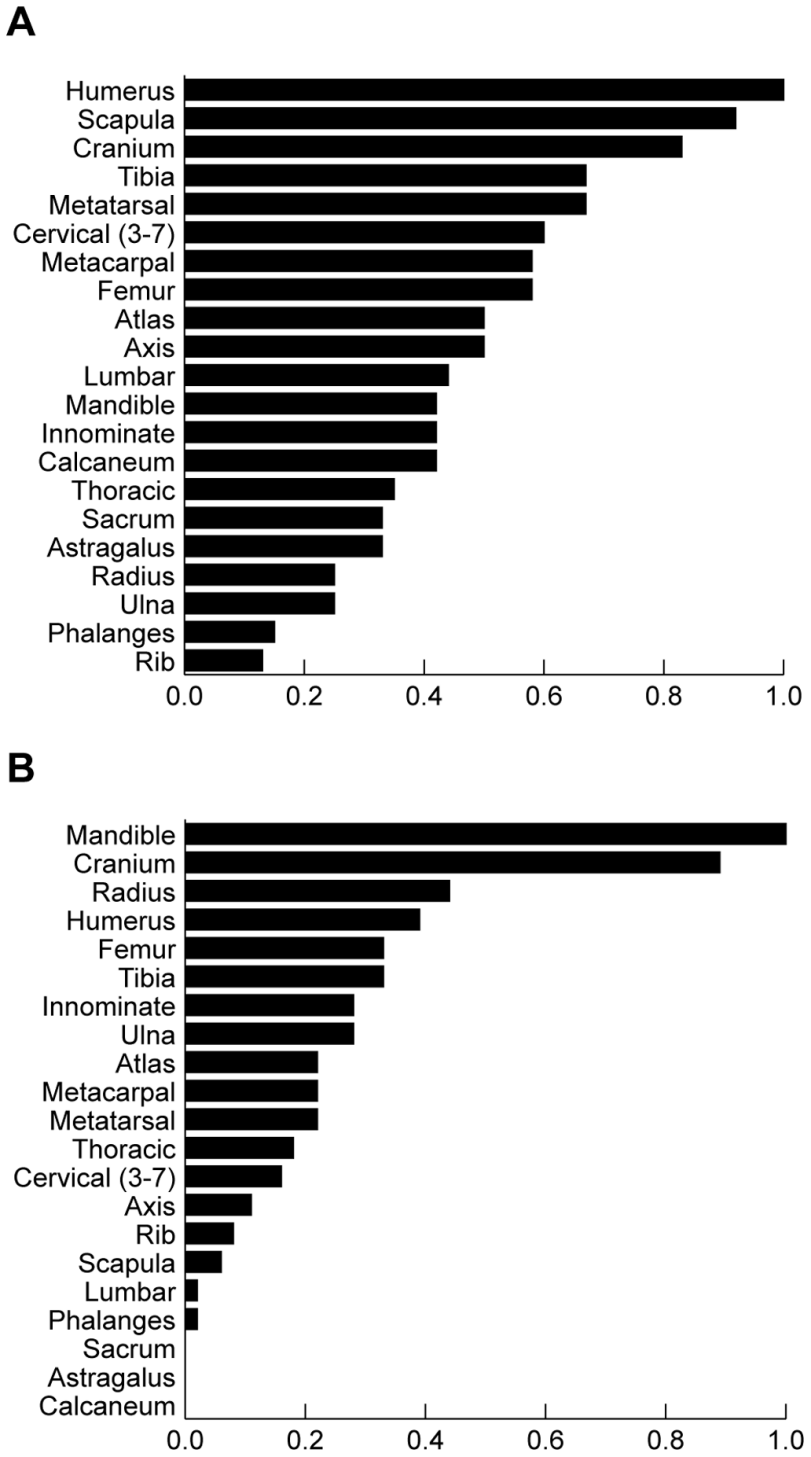
Skeletal element representation for (A) small and (B) medium-sized bovids, Bed KS-1. Abundance data presented as percent minimum animal units (%MAU), calculated following the literature [43]. KJS data derived from Table S3. 100% MAU = 6 for small bovids, 9 for medium-sized bovids. Similar patterns of skeletal element representation are present in Beds KS-2 and KS-3.

Skeletal element analyses further detail the nature of hominin involvement with animal remains (Table S3). Smaller-sized bovids are relatively abundant, both with respect to counts of bony elements and numbers of individuals represented. Specimens from all skeletal regions are present in each assemblage, with high-density cranial and appendicular elements predominating (Figure 5A). Lower-density axial elements (e.g., vertebrae, ribs) are also present, though at proportionately lower frequencies. In each assemblage, skeletal element abundances are positively correlated with bone density values (r_s_ range: 0.368 to 0.655; p-values: 0.110 to 0.002) [59], a pattern consistent with scenarios in which scavenging carnivores removed many of the lower-density remains originally present on-site [22], [25], [27], [60], [61] (Table S4). For cranial and long bone specimens (i.e., ‘high-survival elements’ [HSE]) [61], skeletal element abundances are not significantly correlated with either standardized food utility values (r_s_ range: −0.457 to 0.145; all p-values >0.20) [62] or within-bone nutrient values (r_s_ range: −0.618 to 0.505; all p-values >0.10) [28], [29], suggesting relatively little selectivity in hominin transport with respect to these variables (Tables S5–S7). This latter observation is consistent with Shannon evenness values of HSE’s (range: 0.924 to 0.955), which suggest that small bovid carcasses were transported and deposited as relatively complete units (Table S8) [30]. When considered in sum, the pattern of small bovid skeletal part representation is similar across assemblages, and is consistent with scenarios in which numerous relatively complete carcasses were deposited on-site by hominins [22], [27]–[30], [59]–[62]. Coupled with the results of bone surface modification studies, these data reflect an ecological context in which Oldowan hominins had sustained primary access to the meat and organ tissues of a substantial number of small bovids throughout the deposition of all three geological beds: a period spanning hundreds to thousands of years [50].

With respect to the timing of hominin access to these smaller-sized individuals, actualistic studies in a modern East African grassland (the Serengeti) show that small bovid carcasses are, almost without exception, completely consumed by lions and/or hyenas within the first few minutes to hours following death [63]. Given the relative abundance of small bovid carcasses at KJS (Table S3), the relative dearth of carnivore tooth marks on their remains (Table S1), and the inferred rarity of such scavenging opportunities in grassland settings, our results strongly suggest that hominins acquired many of these animals very early in their resource lives (i.e., fairly close to the moment of death). At present, the summed evidence that Oldowan foragers acquired, defleshed, and demarrowed numerous, complete, small bovids throughout the formation of all three assemblages plausibly represents the earliest archaeological record of hominin hunting activities.

The skeletal remains of medium-sized bovids reflect a slightly different taphonomic history. Although specimens from all skeletal regions are represented, cranial remains predominate (Figure 5B). Within each assemblage, skeletal element abundances are positively correlated with bone densities (r_s_ range: 0.401 to 0.666; all p-values <0.10) [59], and HSE abundances are not significantly correlated with either standardized food utility values (r_s_ range: −0457 to −0.241; all p-values >0.20) [62] or within-bone nutrient values (r_s_ range: 0.107 to 0.657; all p-values >0.10) [28], [29]. When considering the sum of surface modification data, Shannon evenness values (range: 0.808 to 0.944), and theoretical considerations of transport behaviors [61], [62], the record from KJS most parsimoniously indicates that Oldowan hominins introduced the partial remains of medium-sized carcasses to the site, with specific foraging behaviors varying with respect to body region (e.g., head versus postcrania) and timing of access to carcasses [63].

The overall taphonomic history of medium-sized postcrania is thus fairly equivalent to that of the smaller-sized carcasses. In both cases, remains are present at abundances that far exceed natural landscape accumulation norms (Table 1), and bone surface modification frequencies and skeletal part analyses indicate that hominins had primary access to soft tissues (Table 2, Figure 3, Figure 4). The evidence is consistent with scenarios in which hominins introduced a relative abundance of fleshed medium-sized postcrania to the site. In contrast to the record of smaller-sized bovids, however, skeletal element representation and element evenness scores suggest an increased measure of hominin selectivity in skeletal part choice and transport decisions when dealing with medium-sized remains (Table S3, Table S8). Long bone elements are fairly numerous relative to axial remains (as measured by %MAU) (Figure 5B, Table S3); and the more proximal limb elements (i.e., humerus, radio-ulna, femur, and tibia) are relatively more abundant than metapodials (Figure 5B, Table S3). This patterning differs substantially from that of the smaller-sized bovids. The latter’s remains are more evenly-distributed across the entire postcranial skeleton (HSE’s+low survival elements [LSE’s]), as well as across the six major long bones (Figure 5A, Table S3), and presumably reflects the introduction of numerous, fairly complete small bovids to the site. At issue here: what strategies did hominins follow when selecting and transporting medium-sized remains?

The record is potentially consistent with two main scenarios. In the first, hominins introduce an abundance of compete (or relatively complete) medium-sized carcasses to the site. This follows a ‘food maximizing’ strategy in which hominins face negligible-to-minor transport constraints and transfer most or all of the edible remains from death sites to KJS [75]. As a result, they treat both small and medium-sized bovids in a relatively similar manner when making carcass transport decisions. Observed differences in skeletal element records on-site (smalls vs. mediums) would then presumably reflect systematic differences in post-depositional carnivore scavenging behaviors. In the second scenario, hominins preferentially transport limb remains from medium-sized carcasses, plus some axial elements whenever possible. This follows a ‘weight minimizing’ strategy in which transport constraints (e.g., the number of available carriers, distance to destination, predation risk, etc.) limit hominins to carrying away only a subset of all edible tissues [75]. In this case, carnivore treatment of skeletal remains on-site would be relatively consistent across size groups [25], and observed differences in the skeletal element record (small vs. medium) would instead predominantly reflect systematic size-based differences in hominin transport practices.

Here, comparisons between size groups are particularly informative. For small bovids, LSE values are not grossly disproportionate to those of HSE’s (Figure 5A, Table S3). In fact, their overall skeletal record corresponds fairly well to expectations for dual-patterned hominin-first assemblages, [22], [25], [27], [29]. Note too that skeletal remains of smaller-sized individuals are usually at far greater risk of destruction than those of medium-sized animals, especially in grassland contexts [43], [63].This makes the latter’s record at KJS all the more interesting. In each of the assemblages, medium-sized bovids are fairly depauperate in postcranial axial remains relative to both head and limb elements (Figure 5B, Table S3). As the smaller-sized bovids are more evenly represented across the skeleton (both with and without considerations of cranial remains), we discount the possibility that hominins introduced a substantial amount of medium-sized postcranial axial elements to the assemblages (or, alternatively, that those remains were somehow introduced ‘naturally’; e.g., via mass death). In short, if an abundance of medium-sized axial remains were originally present on-site in substantial numbers, and they were largely deleted by scavenging carnivores, then the overall skeletal record of smaller-sized bovids should reflect a substantially more biased record (both in terms of head remains relative to postcrania, and HSE’s relative to LSE’s). The alternative, a null hypothesis in which all bovids were originally present on-site as similarly-apportioned carcasses, would require that medium-sized postcrania (LSE’s+HSE’s) were preferentially deleted by carnivores relative to all smaller-sized remains. We argue that this is unlikely (especially for the record of HSE’s), and note that tooth-mark frequencies are relatively similar across the remains of both size groups (Table S1). In turn, we argue that the KJS record provides robust evidence that hominins largely – but certainly not exclusively – followed a ‘weight-minimizing’ strategy at KJS when selecting and transporting remains from fleshed medium-sized carcasses.

The record of medium-sized cranial elements requires a bit more explanation. Specifically, these remains are disproportionately abundant within each of the assemblages (Figure 5B, Table S3). If hominins largely followed a ‘weight-minimizing’ strategy, and solely had access to complete medium-sized carcasses, they would not have preferentially transported crania and mandibles to KJS. The reason is clear: head remains are quite heavy given their tissue yields, and will often be ignored at death sites in favor of a slew of higher-ranked remains [75]. These same arguments hold when discussing medium-sized limb HSE’s. The preponderance of head remains on-site (as well as the paucity of long bone remains) is thus unlikely to reflect either simple utility or density-related phenomena. Instead, the record strongly suggests the purposeful introduction of a fair number of isolated heads to the site by Oldowan foragers.

But why acquire, transport, and process an abundance of medium-sized heads? In living animals, these remains contain a wealth of fatty, calorie-packed, nutrient-rich tissues: a rare and valuable food resource in a grassland setting where alternate high-value foodstuffs (fruits, nuts, etc.) are often unavailable [2], [3], [29], [49], [52], [63], [76]–[78]. Medium-sized heads are also relatively dense and durable elements, and their internal contents are generally inaccessible to all but hyenas and tool-wielding hominins [63], [79], [80]. As a result, they are often seasonally-available as scavengable resources in East African grasslands [63], [76], [79]–[83]. Additionally, bone surface modification studies at KJS clearly demonstrate that hominins accessed internal head contents: several cranial vault and mandibular fragments bear evidence of percussion striae. Considered in sum, the presumed availability of these isolated remains across the landscape, the relative abundance of these remains in the KJS assemblages, and unambiguous material evidence that hominins exploited their contents on-site is most parsimoniously interpreted as reflecting very early archaeological evidence of a distinct hominin scavenging strategy – one that included a strong focus on acquiring and exploiting fatty, nutrient-rich, energy-dense within-head food resources (e.g., brain matter, mandibular nerve and marrow, etc.) [e.g., 24,63,76,82,84–86].

The total abundance of remains on site, (Table 1), the number of animals represented (Table 1), the high taxonomic diversity present [17], [50], [52], the relatively low frequency of tooth-marked specimens (Figure 3, Figure 4, Table S1), and a sedimentological record wholly inconsistent with a fluvial accumulation of remains [49], [52] also combine to suggest that the KJS assemblages are unlikely to represent *in situ* death or ‘background scatter’ accumulations formed by non-hominin agencies. Similarly, the skeletal element record of medium-sized bovids suggests that they were unlikely to have been present on-site as complete carcasses, an expectation of most ‘kill-site’ and/or landscape accumulation models. When limiting discussion to medium-sized postcrania, the high abundance of limb remains (including many isolated epiphyses) relative to axial elements is also the inverse expectation for landscape assemblages (Figure 5B) [63].

Finally, as with many zooarchaeological assemblages, the KJS skeletal inventories are dominated by numerous unidentifiable long bone shaft fragments. At issue: who or what created these fragments from whole bones? The relative rarity of ‘dry bone’ fractures, coupled with abundant evidence of ‘green bone’ breakage, strongly suggests the involvement of behavioral agents of modification, especially given the inferred low-energy depositional setting at KJS [17], [49]–[52]. Bone surface modifications (e.g., percussion marks and notches; tooth marks and notches) indicative of access to within-bone resources, however, are found at relatively low frequencies in each of the assemblages (Figure 3; Figure 4; Table 2; Table S1; Table S2) [17]. This result is surprising as it is inconsistent with known outcomes of both hominin and carnivore bone breakage practices, where surface modification frequencies are, on average, substantially higher [e.g., 22,23,25,57,58]. A similar pattern of an abundance of shattered but largely unmodified long bone specimens is observed in many other Paleolithic assemblages [31,45,72,73; Table S2], suggesting to us that current bone breakage models may not fully account for all relevant variables. Notably, at KJS there is no evidence that post-depositional sediment compaction and/or bone weathering influenced the bone breakage record [17]. Further experimental research may be required to fully explain these observations.

### Conclusions

The zooarchaeological record from Kanjera South, Kenya provides a rare opportunity to explore early hominin diet and foraging behaviors at ∼2.0 Ma. In each of three large well-preserved faunal assemblages, there is definitive evidence that Oldowan hominins acquired, transported, and consumed the remains of numerous small bovid individuals. Surface modification studies and skeletal element analyses indicate that hominins acquired most or all of these remains relatively early in their resource lives (i.e., in a complete or relatively complete state), providing foragers with access to meat, organ, and within-bone food resources. Given their overall abundance and skeletal representation, unambiguous evidence of their butchery, and their presumed limited availability as potentially-scavengable resources in a grassland setting [17], [27], [63], [87], the small bovid remains at KJS may reflect the earliest archaeological evidence of hominin hunting activities.

The record of medium-sized bovids is slightly more complex. Within each assemblage, there is clear evidence that hominins acquired the postcranial remains of at least some medium-sized individuals relatively early in their resource lives (i.e., with at least some adhering flesh), perhaps mirroring, to some extent, the record of their involvement with smaller-sized bovids [Table S1]. The disproportionate abundance of medium-sized heads, however, likely reflects a separate but complementary foraging activity, one specifically focused on scavenging these remains for their internal food resources (e.g., brain tissues) [17], [63], [84]. This latter portion of the record may represent the earliest archaeological evidence of a distinct hominin scavenging strategy.

With regard to evolutionary ecology, the relative uniformity of hominin activities documented through the KJS sequence indicates an evolved foraging adaptation well-tuned to local ecological contexts. This point implies that hominin involvement with, and their presumed consumption of, animal remains had substantial fitness implications. In turn, sufficiently strong selective pressures are implicated as having favored the evolution of persistent hominin carnivory no later than 2.0 million years ago. This date is approximately 200,000–500,000 years earlier than previously documented [11], [20], [33], [45], and increases the known time depth of this adaptation within the hominin lineage (range of dates reflects varied interpretations of faunal materials from Olduvai [20]–[42]).

Lastly, our findings are directly relevant to a number of interrelated debates within Oldowan hominin paleobiology. These include many of the formative issues of the field, including those that explore the possible relationship(s) between the emergence of persistent hominin carnivory and the evolution of novel social and foraging ecologies [2]–[6], [8], [76], [78], [84], [88]–[90], brain expansion [1]–[5], [19], [78], [88]–[91], range extension [3], [6], [7], [78], [89], life history adaptations [3], [4], [78], [88], [89], [91]–[93], and, potentially, the interplay of some or all of these topics as they relate to the emergence and early evolutionary history of the genus *Homo* [1]–[3], [5]–[7], [78], [88], [89], [91], [92], [94].

## Supporting Information

Table S1 **Bone surface modifications.** Modifications detailed by long bone portion, bed, animal size group, and analyst.
(DOC)

Table S2 **East African Earlier Stone Age zooarchaeological assemblages.** Surface modification data for bovid and taxonomically-indeterminate long bone specimens.
(DOC)

Table S3 **Minimum number of elements (MNE) for small and medium-sized bovids.**
(DOC)

Table S4 **Skeletal element abundances and bone mineral densities.**
(DOC)

Table S5 **Skeletal element abundances and standardized food utility indices.**
(DOC)

Table S6 **Skeletal element abundances and within-bone nutrients.**
(DOC)

Table S7 **Skeletal element abundances and within-bone resource extraction rates.**
(DOC)

Table S8 **Skeletal element evenness.** Evenness calculated using the Shannon evenness index. Bone frequencies modeled using a Bayesian multinomial model.
(DOC)
