# Supplementary material for: Earliest Archaeological Evidence of Persistent Hominin Carnivory
Source: PLoS One. 2013 Apr 25;8(4):e62174. doi: 10.1371/journal.pone.0062174 (PMC3636145; doi:10.1371/journal.pone.0062174)
Supplement: Table S1 — Bone surface modifications. Modifications detailed by long bone portion, bed, animal size group, and analyst. (DOC) [file pone.0062174.s001.doc]

**Table S1. Bone surface modifications**

|  |  | **Epiphyseal Fragments** | | | |  | **Near-Epiphyseal Fragments** | | | |
| --- | --- | --- | --- | --- | --- | --- | --- | --- | --- | --- |
| **Bed** | **Body Size** | **TM** | **CM** | **PM** | **N** | **Bed** | **TM** | **CM** | **PM** | **N** |
| KS-1 | Small | 5,6,4 | 0,0,0 | 0,0,0 | 24 | KS-1 | 1,1,1 | 0,0,0 | 0,0,1 | 5 |
|  | % | 20.8, 25.0, 16.7 | 0, 0, 0 | 0, 0, 0 |  |  | 20.0, 20.0, 20.0 | 0, 0, 0 | 0, 0, 20.0 |  |
|  | Medium | 1,2,2 | 1,1,1 | 0,0,0 | 10 |  | 2,2,2 | 0,0,0 | 0,0,0 | 6 |
|  | % | 10.0, 20.0, 20.0 | 10.0, 10.0, 10.0 | 0, 0, 0 |  |  | 33.3, 33.3, 33.3 | 0, 0, 0 | 0, 0, 0 |  |
|  | Size x | - | - | - | 0 |  | 0,1,0 | 0,1,1 | 2,1,4 | 15 |
|  | % | - | - | - |  |  | 0, 6.7, 0 | 0, 6.7, 6.7 | 13.3, 6.7, 26.7 |  |
|  | Sum | 6,8,6 | 1,1,1 | 0,0,0 | 34 |  | 3,4,3 | 0,1,1 | 2,1,5 | 26 |
|  | % | 17.6, 23.5, 17.6 | 2.9, 2.9, 2.9 | 0, 0, 0 |  |  | 11.5, 15.4, 11.5 | 0, 3.8, 3.8 | 7.7, 3.8, 19.2 |  |
|  | Sum min | 4 (11.8 %) | 1 (2.9%) | 0 (0%) | 34 |  | 3 (11.5%) | 0 (0%) | 1 (3.8%) | 26 |
|  | Sum max | 11 (32.4%) | 1 (2.9%) | 0 (0%) | 34 |  | 4 (15.4%) | 1 (3.8%) | 5 (19.2%) | 26 |
| KS-2 | Small | 4,6,3 | 4,3,4 | 0,1,1 | 30 | KS-2 | 0,3,3 | 0,1,2 | 0,0,1 | 16 |
|  | % | 13.3, 20.0, 10.0 | 13.3, 10.0, 13.3 | 0, 3.3, 3.3 |  |  | 0, 18.8, 18.8 | 0, 6.3, 12.5 | 0, 0, 6.3 |  |
|  | Medium | 4,4,3 | 0,2,3 | 2,3,3 | 32 |  | 2,2,2 | 0,0,0 | 0,0,0 | 14 |
|  | % | 12.5, 12.5, 9.4 | 0, 6.3, 9.4 | 6.3, 9.4, 9.4 |  |  | 14.3, 14.3, 14.3 | 0, 0, 0 | 0, 0, 0 |  |
|  | Size x | 0,1,1 | 0,0,0 | 0,1,1 | 2 |  | 1,3,3 | 1,2,3 | 0,0,1 | 24 |
|  | % | 0, 50.0, 50.0 | 0, 0, 0 | 0, 50.0, 50.0 |  |  | 4.2, 12.5, 12.5 | 4.2, 8.3, 12.5 | 0, 0, 4.2 |  |
|  | Sum | 8,11,7 | 4,5,7 | 2,5,5 | 64 |  | 3,8,8 | 1,3,5 | 0,0,2 | 54 |
|  | % | 12.5, 17.2, 10.9 | 6.3, 7.8, 10.9 | 3.1, 7.8, 7.8 |  |  | 5.6, 14.8, 14.8 | 1.9, 5.6, 9.3 | 0, 0, 3.7 |  |
|  | Sum min | 4 (6.3%) | 3 (4.7%) | 2 (3.1%) | 64 |  | 3 (5.6%) | 1 (1.9%) | 0 (0%) | 54 |
|  | Sum max | 12 (18.8%) | 7 (10.9%) | 7 (10.9%) | 64 |  | 12 (22.2%) | 5 (9.3%) | 2 (3.7%) | 54 |
| KS-3 | Small | 0,0,0 | 0,0,0 | 0,0,0 | 8 | KS-3 | 0,0,0 | 0,0,0 | 1,1,1 | 2 |
|  | % | 0, 0, 0 | 0, 0, 0 | 0, 0, 0 |  |  | 0, 0, 0 | 0, 0, 0 | 50.0, 50.0, 50.0 |  |
|  | Medium | 1,1,0 | 0,0,0 | 0,0,0 | 3 |  | 0,0,0 | 2, 1, 2 | 0,0,0 | 2 |
|  | % | 33.3, 33.3, 0 | 0, 0, 0 | 0, 0, 0 |  |  | 0, 0, 0 | 100, 50, 100 | 0, 0, 0 |  |
|  | Size x | - | - | - | 0 |  | 0,0,0 | 1,1,1 | 0,0,0 | 5 |
|  | % | - | - | - |  |  | 0, 0, 0 | 20.0, 20.0, 20.0 | 0, 0, 0 |  |
|  | Sum | 1,1,0 | 0,0,0 | 0,0,0 | 11 |  | 0,0,0 | 3,2,3 | 1,1,1 | 9 |
|  | % | 9.1, 9.1, 0 | 0, 0, 0 | 0, 0, 0 |  |  | 0, 0, 0 | 33.3, 22.2, 33.3 | 11.1, 11.1, 11.1 |  |
|  | Sum min | 0 (0%) | 0 (0%) | 0 (0%) | 11 |  | 0 (0%) | 2 (22.2%) | 1 (11.1%) | 9 |
|  | Sum max | 1 (9.1%) | 0 (0%) | 0 (0%) | 11 |  | 0 (0%) | 3 (33.3%) | 1 (11.1%) | 9 |
| Sum | Small | 9,12,7 | 4,3,4 | 0,1,1 | 62 | Sum | 1,4,4 | 0,1,2 | 1,1,3 | 23 |
|  | % | 14.5, 19.4, 11.3 | 6.5, 4.8, 6.5 | 0, 1.6, 1.6 |  |  | 4.3, 17.4, 17.4 | 0, 4.3, 8.7 | 4.3, 4.3, 13.0 |  |
|  | Medium | 6,7,5 | 1,3,4 | 2,3,3 | 45 |  | 4,4,4 | 2,1,2 | 0,0,0 | 22 |
|  | % | 13.3, 15.6, 11.1 | 2.2, 6.7, 8.9 | 4.4, 6.7, 6.7 |  |  | 18.2, 18.2, 18.2 | 9.1, 4.5, 9.1 | 0, 0, 0 |  |
|  | Size x | 0,1,1 | 0,0,0 | 0,1,1 | 2 |  | 1,4,3 | 2,4,5 | 2,1,5 | 44 |
|  | % | 0, 50.0, 50.0 | 0, 0, 0 | 0, 50.0, 50.0 |  |  | 2.3, 9.1, 6.8 | 4.5, 9.1, 11.4 | 4.5, 2.3, 11.4 |  |
|  | Sum | 15,20,13 | 5,6,8 | 2,5,5 | 109 |  | 6,12,11 | 4,6,9 | 3,2,8 | 89 |
|  | % | 13.8, 18.3, 11.9 | 4.6, 5.5, 7.3 | 1.8, 4.6, 4.6 |  |  | 6.7, 13.5, 12.4 | 4.5, 6.7, 10.1 | 3.4, 2.2, 9.0 |  |
|  | Sum min | 8 (7.3%) | 4 (3.7%) | 2 (1.8%) | 109 |  | 6 (6.7%) | 3 (3.4%) | 2 (2.2%) | 89 |
|  | Sum max | 24 (22.0%) | 8 (7.3%) | 7 (6.4%) | 109 |  | 16 (18.0%) | 9 (10.1%) | 8 (9.0%) | 89 |

|  |  | **Mid-Shaft Fragments** | | | |  | **Sum** | | | |
| --- | --- | --- | --- | --- | --- | --- | --- | --- | --- | --- |
| **Bed** | **Body Size** | **TM** | **CM** | **PM** | **N** | **Bed** | **TM** | **CM** | **PM** | **N** |
| KS-1 | Small | 2,2,2 | 0,0,0 | 1,1,1 | 15 | KS-1 | 8,9,7 | 0,0,0 | 1,1,2 | 44 |
|  | % | 13.3, 13.3, 13.3 | 0, 0, 0 | 6.7, 6.7, 6.7 |  |  | 18.2, 20.5, 15.9 | 0, 0, 0 | 2.3, 2.3, 4.5 |  |
|  | Medium | 1,1,1 | 1,1,1 | 2,2,3 | 13 |  | 4,5,5 | 2,2,2 | 2,2,3 | 29 |
|  | % | 7.7, 7.7, 7.7 | 7.7, 7.7, 7.7 | 15.4, 15.4, 23.1 |  |  | 13.8, 17.2, 17.2 | 6.9, 6.9, 6.9 | 6.9, 6.9, 10.3 |  |
|  | Size x | 5,7,5 | 1,3,3 | 6,6,6 | 68 |  | 5,8,5 | 1,4,4 | 8,7,10 | 83 |
|  | % | 7.4, 10.3, 7.4 | 1.5 4.4, 4.4 | 8.8, 8.8, 8.8 |  |  | 6.0, 9.6, 6.0 | 1.2, 4.8, 4.8 | 9.6, 8.4, 12.0 |  |
|  | Sum | 8,10,8 | 2,4,4 | 9, 9, 10 | 96 |  | 17,22,17 | 3,6,6 | 11,10,15 | 156 |
|  | % | 8.3, 10.4, 8.3 | 2.1, 4.2, 4.2 | 9.4, 9.4, 10.4 |  |  | 10.9, 14.1, 10.9 | 1.9, 3.8, 3.8 | 7.1, 6.4, 9.6 |  |
|  | Sum min | 5 (5.2%) | 2 (2.1%) | 7 (7.3%) | 96 |  | 12 (7.7%) | 3 (1.9%) | 8 (5.1%) | 156 |
|  | Sum max | 12 (12.5%) | 5 (5.2%) | 13 (13.5%) | 96 |  | 27 (17.3%) | 7 (4.5%) | 18 (11.5%) | 156 |
| KS-2 | Small | 3,4,4 | 0,0,0 | 0,0,0 | 28 | KS-2 | 7,13,10 | 4,4,6 | 0,1,2 | 74 |
|  | % | 10.7, 14.3, 14.3 | 0, 0, 0 | 0, 0, 0 |  |  | 9.5, 17.6, 13.5 | 5.4,5.4, 8.1 | 0, 1.4, 2.7 |  |
|  | Medium | 1,3,2 | 0,1,3 | 1,2,6 | 33 |  | 7,9,7 | 0,3,6 | 3,5,9 | 79 |
|  | % | 3.0, 9.1, 6.1 | 0, 3.0, 9.1 | 3.0, 6.1, 18.2 |  |  | 8.9, 11.4, 8.9 | 0, 3.8, 7.6 | 3.8, 6.3, 11.4 |  |
|  | Size x | 15,25,18 | 2,5,3 | 7,8,10 | 146 |  | 16,29,22 | 3,7,6 | 7,9,12 | 172 |
|  | % | 10.3, 17.1, 12.3 | 1.4, 3.4, 2.1 | 4.8, 5.5, 6.8 |  |  | 9.3, 16.9, 12.8 | 1.7, 4.1, 3.5 | 4.1, 5.2, 7.0 |  |
|  | Sum | 19,32,24 | 2,6,6 | 8,10,16 | 207 |  | 30,51,39 | 7,14,18 | 10,15,23 | 325 |
|  | % | 9.2, 15.5, 11.6 | 1.0, 2.9, 2.9 | 3.9, 4.8, 7.7 |  |  | 9.2, 15.7, 12.0 | 2.2, 4.3, 5.5 | 3.1, 4.6, 7.1 |  |
|  | Sum min | 14 (6.8%) | 2 (0.5%) | 5 (2.4%) | 207 |  | 21 (6.5%) | 6 (1.9%) | 7 (2.2%) | 325 |
|  | Sum max | 39 (18.8%) | 9 (4.3%) | 21 (10.1%) | 207 |  | 63 (19.4%) | 21 (6.5%) | 30 (9.2%) | 325 |
| KS-3 | Small | 0,1,0 | 0,0,0 | 0,0,2 | 5 | KS-3 | 0,1,0 | 0,0,0 | 1,1,3 | 15 |
|  | % | 0, 20.0, 0 | 0, 0, 0 | 0, 0, 40.0 |  |  | 0, 6.7, 0 | 0, 0, 0 | 6.7, 6.7, 20.0 |  |
|  | Medium | 1,1,0 | 0, 0, 0 | 0,0,1 | 1 |  | 2,2,0 | 2,1,2 | 0,0,1 | 6 |
|  | % | 100, 100, 0 | 0, 0, 0 | 0, 0, 100 |  |  | 33,3, 33.3, 0 | 33.3, 16.7, 33.3 | 0, 0, 16.7 |  |
|  | Size x | 1,3,1 | 1,0,0 | 2,1,3 | 38 |  | 1,3,1 | 2,1,1 | 2,1,3 | 43 |
|  | % | 2.6, 7.9, 2.6 | 2.6, 0, 0 | 5.3, 2.6, 7.9 |  |  | 2.3, 7.0, 2.3 | 4.7, 2.3, 2.3 | 4.7, 2.3, 7.0 |  |
|  | Sum | 2,5,1 | 1,0,0 | 2,1,6 | 44 |  | 3,6,1 | 4,2,3 | 3,2,7 | 64 |
|  | % | 4.6, 11.4, 2.3 | 2.3, 0, 0 | 4.6, 2.3, 13.6 |  |  | 4.7, 9.4, 1.6 | 6.3, 3.1, 4.7 | 4.7, 3.1, 10.9 |  |
|  | Sum min | 1 (2.3%) | 0 (0%) | 0 (0%) | 44 |  | 1 (1.6%) | 2 (3.1%) | 1 (1.6%) | 64 |
|  | Sum max | 5 (11.4%) | 1 (2.3%) | 7 (15.9%) | 44 |  | 6 (9.4%) | 4 (6.3%) | 8 (12.5%) | 64 |
| Sum | Small | 5,7,6 | 0,0,0 | 1,1,3 | 48 | Sum | 15,23,17 | 4,4,6 | 2,3,7 | 133 |
|  | % | 10.4, 14.6, 12.5 | 0, 0, 0 | 2.1, 2.1, 6.3 |  |  | 11.3, 17.3, 12.8 | 3.0, 3.0, 4.5 | 1.5, 2.3, 5.3 |  |
|  | Medium | 3,5,3 | 1,2,4 | 3,4,10 | 47 |  | 13,16,12 | 4,6,10 | 5,7,13 | 114 |
|  | % | 6.4, 10.6, 6.4 | 2.1, 4.3, 8.5 | 6.4, 8.5, 21.3 |  |  | 11.4, 14.0, 10.5 | 3.5, 5.3, 8.8 | 4.4, 6.1, 11.4 |  |
|  | Size x | 21,35,24 | 4,8,6 | 15,15,19 | 252 |  | 22,40,28 | 6,12,11 | 17,17,25 | 298 |
|  | % | 8.3, 13.9, 9.5 | 1.6, 3.2, 2.4 | 6.0, 6.0, 7.5 |  |  | 7.4, 13.4, 9.4 | 2.0, 4.0, 3.7 | 5.7, 5.7, 8.4 |  |
|  | Sum | 29,47,33 | 5,10,10 | 19,20,32 | 347 |  | 50,79,57 | 14,22,27 | 24,27,45 | 545 |
|  | % | 8.4, 13.5, 9.5 | 1.4, 2.9, 2.9 | 5.5, 5.8, 9.2 |  |  | 9.2, 14.5, 10.5 | 2.6, 4.0, 5.0 | 4.4, 5.0, 8.3 |  |
|  | Sum min | 20 (5.8%) | 4 (1.2%) | 12 (3.5%) | 347 |  | 34 (6.2%) | 11 (2.0) | 16 (2.9%) | 545 |
|  | Sum max | 56 (16.1%) | 15 (4.3%) | 41 (11.8%) | 347 |  | 96 (17.6%) | 32 (5.9%) | 56 (10.3%) | 545 |

**Table S1.** Modifications detailed by long bone portion [1-3], bed, animal size group, and analyst. Epiphyseal fragments (EPI) possess at least some of the proximal or distal articular surface. Near-epiphyseal fragments (NEF) lack an epiphyseal joint surface, but have at least some cancellous bone, usually on the medullary surface. Mid-shaft fragments (MSH) are diaphyseal specimens that lack cancellous bone on medullary surfaces. Body sizes follow Bunn [4], with bovids of sizes 1 and 2 considered ‘small’, and those of 2/3a and 3 considered ‘medium-sized’. When unable to confidently attribute a bone fragment to size 2, 2/3a, or 3, fossils are coded as ‘size x’. ‘Sum min’ is the sum of specimens unanimously identified by all three analysts as bearing a given class of modification. ‘Sum max’ is the sum of specimens identified by one or more analysts as bearing a given class of modification. Bone modifications follow the literature (5 and references therein), and include tooth marks (TM: pits, scores, furrows), cut marks (CM), and percussion marks (PM: pits, striae). Bone modification frequencies are listed by analyst: Ferraro, Pobiner, and Oliver (in order from left to right). Samples are bovid and taxonomically-indeterminate long bone specimens (i.e., humerus, radius, metacarpal, femur, tibia, metatarsal, or ‘long bone shaft fragment’), > 2cm in length, from body sizes 1-3, with ‘very good’ to ‘excellent’ bone surface preservation (i.e., surface conditions 4 -5 [5]) and without recent or geological fractures. Bone damage values presented here differ slightly from a previous treatment [5] as the current sample is limited to specimens with only the highest quality bone surfaces.

1 Blumenschine RJ, Marean CW (1993) ) A carnivore’s view of archaeological bone assemblages. In: Hudson J, editor. From Bones to Behavior: Ethnoarchaeological and Experimental Contributions to the Interpretation of Faunal Remains. Carbondale: University of Southern Illinois Press. Pp. 273-300.

2 Blumenschine RJ (1995) Percussion marks, tooth marks, and experimental determinations of the timing of hominid and carnivore access to long bones at FLK Zinjanthropus, Olduvai Gorge, Tanzania. J Hum Evol 29: 21-51.

3 Capaldo SD (1997) Experimental determinations of carcass processing by Plio-Pleistocene hominids and carnivores at FLK 22 (Zinjanthropus), Olduvai Gorge, Tanzania. J Hum Evol 33: 555-597.

4 Bunn HT (1982) Meat-eating and human evolution: studies on the diet and subsistence patterns of Plio-Pleistocene hominids in East Africa [PhD dissertation]. Berkeley: University of California, Berkeley.

5 Ferraro JV (2007) Broken bones and shattered stones: on the foraging ecology of Oldowan hominins [PhD dissertation]. Los Angeles: University of California, Los Angeles.
