# Supplementary material for: Earliest Archaeological Evidence of Persistent Hominin Carnivory
Source: PLoS One. 2013 Apr 25;8(4):e62174. doi: 10.1371/journal.pone.0062174 (PMC3636145; doi:10.1371/journal.pone.0062174)
Supplement: Table S2 — East African Earlier Stone Age zooarchaeological assemblages. Surface modification data for bovid and taxonomically-indeterminate long bone specimens. (DOC) [file pone.0062174.s002.doc]

**Table S2. East African Earlier Stone Age zooarchaeological assemblages**

| **Date (Ma)** | **Sizes** | **Assemblage** | **Analyst(s)** | **TM %** | **CM %** | **PM %** | **N** |
| --- | --- | --- | --- | --- | --- | --- | --- |
| 2.0 | 1-3 (all) | KS-1 | JVF | 10.9 | 1.9 | 7.1 | 156 |
| 2.0 | 1-3 (all) | KS-1 | BLP | 14.1 | 3.8 | 6.4 | 156 |
| 2.0 | 1-3 (all) | KS-1 | JSO | 10.9 | 3.8 | 9.6 | 156 |
| 2.0 | 1-3 (all) | KS-2 | JVF | 9.2 | 2.2 | 3.1 | 325 |
| 2.0 | 1-3 (all) | KS-2 | BLP | 15.7 | 4.3 | 4.6 | 325 |
| 2.0 | 1-3 (all) | KS-2 | JSO | 12.0 | 5.5 | 7.1 | 325 |
| 2.0 | 1-3 (all) | KS-3 | JVF | 4.7 | 6.3 | 4.7 | 64 |
| 2.0 | 1-3 (all) | KS-3 | BLP | 9.4 | 3.1 | 3.1 | 64 |
| 2.0 | 1-3 (all) | KS-3 | JSO | 1.6 | 4.7 | 10.9 | 64 |
| 1.2 | 1-4 (all) | BK | CMM [5] | 4.8 | - | - | 809 |
| 1.2 | 1-4 (6lb) | BK | CMM (raw) [5] | 5.3 | 5.5 | 7.8 | 528 |
| 1.2 | 1-4 (6lb) | BK | CPE+MDR [6] | 12.0 | 2.8 | 2.5 | 683 |
| 1.2 | 1-3 (6lb) | BK | CPE+MDR [6] | 12.8 | 3.1 | 3.1 | 553 |
| 1.2 | 1-3 (all) | BK | CPE+MDR [6] | 10.7 | 2.4 | 2.2 | 878 |
| 1.2 | 1-5 (ms) | BK1 | MDR et al. [7] | 8.3 | 18.5 | 32.4 | 108 |
| 1.2 | 1-5 (ms) | BK2 | MDR et al. [7] | 8.8 | 20.5 | 25.4 | 204 |
| 1.2 | 1-5 (ms) | BK3 | MDR et al. [7] | 2.3 | 17.9 | 27.3 | 128 |
| 1.2 | 1-5 (ms) | BK4 | MDR et al. [7] | 5.5 | 11.0 | 11.0 | 18 |
| 1.5 | 1-4 (all) | FwJj 14a | BLP+CMM+MJR [8] | 1.2 | 16.9 | 3.6 | 331 |
| 1.5 | 1-4 (all) | FwJj 14b | BLP+CMM+MJR [8] | 0 | 23.0 | 5.8 | 139 |
| 1.5 | 1-4 (all) | GaJi 14 | BLP+CMM+MJR [8] | 0* | 16.7 | 2.7 | 257 |
| 1.6 | 1-4 (all) | FxJj 50 | HTB [9] | - | 2.9 | - | 278 |
| 1.6 | 1-4 (all) | FxJj 50 | MDR[10] | 35.7 | 12.7 | 9.5 | 126 |
| 1.8 | 1-4 (all) | FLK 22 | MDR+RB [11] | 14.9 | - | 15.1 | 696 |
| 1.8 | 1-4 (all) | FLK 22 | RJB [3,4] | 60.7 | 18.7 | 27.4 | 731 |
| 1.8 | 1-4 (all) | FLK 22 | HTB+EMK (micro) [12] | - | 7.9 | - | 1432 |
| 1.8 | 1-5 (all) | FLK 22 | JSO [13] | 16.8 | 12.2 | - | 1914 |

**Table S2.** Surface modification data for bovid and taxonomically-indeterminate long bone specimens. KJS data from table S1 for summed long bone portions and body sizes. Bone modification terms and definitions [1-4] include tooth marks (TM: pits, scores, furrows), cut marks (CM), and percussion marks (PM: pits, striae). As few size 4 (i.e., large animal) remains have been recovered from KJS, we limit our analyses to summed sizes 1-3 (i.e., small and medium-sized individuals). Given the tiny sample of size 4 remains, KJS bone surface modification frequencies are virtually identical for summed sizes 1-3 and 1-4 remains. All assemblages include bovid and taxonomically-indeterminate long bone specimens: with most assemblages further limited to specimens > 2cm in length, with good bone surface preservation, and without recent or geological fractures. Data from BK are available in three formats: ‘all’, ‘6lb’, and ‘ms’. ‘All’ includes long bone shaft fragments not identified to element or size, ‘6lb’ includes only those specimens identified to one of the 6 major long bones. The ‘ms’ subset solely contains well-preserved mid-shaft specimens without ‘dry breaks’. Data for BK from [5-7]; FwJj14a, FwJj14b, GaJi14 from [8]; FxJj50 from [9-10]; FLK22 from [3-4,11-13]. ‘Raw’ [5] and ‘micro’ [12] are published data subsets. Where analysts are grouped by ‘+’, two or more analysts provide a single interpretation of surface modifications. * = assemblage GaJi14 has a single crocodile tooth-marked specimen [8].

1 Bunn HT (1982) Meat-eating and human evolution: studies on the diet and subsistence patterns of Plio-Pleistocene hominids in East Africa [PhD dissertation]. Berkeley: University of California, Berkeley.

2 Blumenschine RJ, Marean CW (1993) A carnivore’s view of archaeological bone assemblages. In: Hudson J, editor. From Bones to Behavior: Ethnoarchaeological and Experimental Contributions to the Interpretation of Faunal Remains. Carbondale: University of Southern Illinois Press. Pp. 273-300.

3 Blumenschine RJ (1995) Percussion marks, tooth marks, and experimental determinations of the timing of hominid and carnivore access to long bones at FLK *Zinjanthropus*, Olduvai Gorge, Tanzania. J Hum Evol 29: 21-51.

4 Capaldo SD (1997) Experimental determinations of carcass processing by Plio-Pleistocene hominids and carnivores at FLK 22 (*Zinjanthropus*), Olduvai Gorge, Tanzania. J Hum Evol 33: 555-597.

5 Monahan CM (1996) New zooarchaeological data from Bed II, Olduvai Gorge, Tanzania: implications for hominid behavior in the Early Pleistocene. J Hum Evol 31: 93-128.

6 Egeland CP, Dominguez-Rodrigo M (2008) Taphonomic perspectives on hominid site use and foraging strategies during Bed II times at Olduvai Gorge, Tanzania. J Hum Evol 55: 1031-1052.

7 Dominguez-Rodrigo M, Mabulla A, Bunn HT, Barba R, Diez-Martin F, et al. (2009) Unravelling hominin behavior at another anthropogenic site from Olduvai Gorge (Tanzania): new archaeological and taphonomic research at BK, Upper Bed II. J Hum Evol 57: 260-283.

8 Pobiner BL, Rogers MJ, Monahan CM, Harris JWK (2008) New evidence for hominin carcass processing strategies at 1.5 Ma, Koobi Fora, Kenya. J Hum Evol 55: 103-130.

9 Bunn HT (1997) The bone assemblages from the excavated sites. In: Isaac GL, editor. Koobi Fora Research Project Volume 5: Plio-Pleistocene Archaeology. Oxford: Clarendon Press. pp. 402-458.

10 Dominguez-Rodrigo M (2002) Hunting and scavenging by early humans: the state of the debate. Journal of World Prehistory 16: 1-54.

11 Dominguez-Rodrigo M, Barba R (2006) New estimates of tooth mark and percussion mark frequencies at the FLK *Zinj* site: the carnivore-hominid-carnivore hypothesis falsified. J Hum Evol 50: 170-194.

12 Bunn HT, Kroll EM (1986) Systematic butchery by Plio/Pleistocene hominids at Olduvai Gorge, Tanzania. Curr Anthropol 27: 431-452.

13 Oliver JS (1994) Estimates of hominid and carnivore involvement in the FLK *Zinjanthropus* fossil assemblage: some socioecological implications. J Hum Evol 27: 267-294.
