# Supplementary material for: Earliest Archaeological Evidence of Persistent Hominin Carnivory
Source: PLoS One. 2013 Apr 25;8(4):e62174. doi: 10.1371/journal.pone.0062174 (PMC3636145; doi:10.1371/journal.pone.0062174)
Supplement: Table S3 — Minimum number of elements (MNE) for small and medium-sized bovids. (DOC) [file pone.0062174.s003.doc]

**Table S3. Minimum number of elements (MNE) for small and medium-sized bovids**

|  | **KS-1 Small** | **KS-1 Medium** | **KS-2 Small** | **KS-2 Medium** | **KS-3 Small** | **KS-3 Medium** |
| --- | --- | --- | --- | --- | --- | --- |
| Cranium | 5 | 8 | 5 | 14 | 3 | 6 |
| Hemimandible | 5 | 18 | 21 | 21 | 2 | 7 |
| Atlas | 3 | 2 | 0 | 4 | 1 | 0 |
| Axis | 3 | 1 | 1 | 0 | 0 | 0 |
| Cervical (3-7) | 18 | 7 | 4 | 7 | 0 | 4 |
| Thoracic | 27 | 21 | 8 | 9 | 4 | 3 |
| Lumbar | 16 | 1 | 7 | 3 | 3 | 1 |
| Sacrum | 2 | 0 | 0 | 0 | 0 | 0 |
| Innominate | 5 | 5 | 13 | 5 | 3 | 1 |
| Rib | 21 | 19 | 20 | 22 | 4 | 4 |
| Scapula | 11 | 1 | 13 | 4 | 2 | 1 |
| Humerus | 12 | 7 | 13 | 13 | 1 | 3 |
| Radius | 3 | 8 | 9 | 16 | 3 | 3 |
| Ulna | 3 | 5 | 4 | 4 | 2 | 0 |
| Metacarpal | 7 | 4 | 9 | 8 | 5 | 0 |
| Femur | 7 | 6 | 8 | 7 | 3 | 1 |
| Tibia | 8 | 6 | 6 | 15 | 3 | 1 |
| Astragalus | 4 | 0 | 9 | 2 | 2 | 0 |
| Calcaneum | 5 | 0 | 3 | 2 | 0 | 1 |
| Metatarsal | 8 | 4 | 13 | 9 | 3 | 1 |
| Phalanges | 22 | 4 | 17 | 5 | 4 | 2 |

**Table S3.** MNE calculations made following the ‘refitting method’ [e.g., 1-5], which places a strong emphasis on shaft portions for long bones, and incorporates taxonomy, age, and size in calculations where appropriate. Small bovids are body sizes 1 and 2; medium-sized bovids are body sizes 2/3a to 3b [6]. Numbers differ slightly from a previous treatment [2], as size 2/3a elements are grouped with ‘medium-sized’ remains in this paper.

1. Lyman RL (1994) Vertebrate Taphonomy. Cambridge: Cambridge University Press.

2. Ferraro JV (2007) Broken bones and shattered stones: on the foraging ecology of Oldowan hominins [PhD dissertation]. Los Angeles: University of California, Los Angeles.

3. Egeland CP, Dominguez-Rodrigo M (2008) Taphonomic perspectives on hominid site use and foraging strategies during Bed II times at Olduvai Gorge, Tanzania. J Hum Evol 55: 1031-1052.

4. Bunn HT, Kroll EM (1986) Systematic butchery by Plio/Pleistocene hominids at Olduvai Gorge, Tanzania. Curr Anthropol 27: 431-452.

5. Marean CW, Abe Y, Frey CJ, Randall RC (2000) Zooarchaeological and taphonomic analysis of the Die Kelders Cave 1 Layers 10 and 11 Middle Stone Age larger mammal fauna. J Hum Evol 38: 197-233.

6. Bunn HT (1982) Meat-eating and human evolution: studies on the diet and subsistence patterns of Plio-Pleistocene hominids in East Africa [PhD dissertation]. Berkeley: University of California, Berkeley.
