# Supplementary material for: Earliest Archaeological Evidence of Persistent Hominin Carnivory
Source: PLoS One. 2013 Apr 25;8(4):e62174. doi: 10.1371/journal.pone.0062174 (PMC3636145; doi:10.1371/journal.pone.0062174)
Supplement: Table S4 — Skeletal element abundances and bone mineral densities. (DOC) [file pone.0062174.s004.doc]

**Table S4. Skeletal element abundances and bone mineral densities.**

| **Bed** | **Body size** | **N** | **Spearman's rho** | **Significance (2-tailed)** |
| --- | --- | --- | --- | --- |
| KS-1 | Small | 20 | 0.368 | 0.110 |
|  | Medium | 20 | **0.576** | **0.008** |
| KS-2 | Small | 20 | **0.648** | **0.002** |
|  | Medium | 20 | **0.666** | **0.001** |
| KS-3 | Small | 20 | **0.655** | **0.002** |
|  | Medium | 20 | 0.401 | 0.080 |

**Table S4.** Correlation coefficients (r_s_) between skeletal element abundances and bone mineral densities. Skeletal element abundance data (minimum animal units; MAU [1]) derived from table S3; cranium not included. We used published bone density values for *Connochaetes taurinus* (wildebeest) [2]. Whenever possible, we used the maximum BMD_2_ value for each element. For bones without BMD_2_ data, we used the maximum BMD_1_ value by element. Analyses were run separately for small and medium-sized bovids. Results in bold are significant at the .01 level (2-tailed).

1. Lyman RL (1994) Vertebrate Taphonomy. Cambridge: Cambridge University Press.

2. Lam YM, Chen X, Pearson OM (1999) Intertaxonomic variability in patterns of bone density and the differential representation of bovid, cervid, and equid elements in the archaeological record. Am Antiq 64: 343-362.
