# Supplementary material for: Earliest Archaeological Evidence of Persistent Hominin Carnivory
Source: PLoS One. 2013 Apr 25;8(4):e62174. doi: 10.1371/journal.pone.0062174 (PMC3636145; doi:10.1371/journal.pone.0062174)
Supplement: Table S5 — Skeletal element abundances and standardized food utility indices. (DOC) [file pone.0062174.s005.doc]

**Table S5. Skeletal element abundances and standardized food utility indices.**

| **Bed** | **Body size** | **Condition** | **N** | **Spearman's rho** | **Significance (2-tailed)** |
| --- | --- | --- | --- | --- | --- |
| KS-1 | Small | With tongue | 8 | 0.145 | 0.733 |
|  |  | W/o tongue |  | 0.120 | 0.776 |
| KS-1 | Medium | With tongue | 8 | -0.241 | 0.565 |
|  |  | W/o tongue |  | -0.289 | 0.487 |
| KS-2 | Small | With tongue | 8 | -0.277 | 0.506 |
|  |  | W/o tongue |  | -0.386 | 0.346 |
| KS-2 | Medium | With tongue | 8 | -0.405 | 0.320 |
|  |  | W/o tongue |  | -0.429 | 0.289 |
| KS-3 | Small | With tongue | 8 | -0.457 | 0.255 |
|  |  | W/o tongue |  | -0.393 | 0.335 |
| KS-3 | Medium | With tongue | 8 | -0.246 | 0.558 |
|  |  | W/o tongue |  | -0.282 | 0.498 |

**Table S5.** Correlation coefficients (r_s_) between skeletal element abundances and standardized food utility indices. Skeletal element abundance data (minimum animal units; MAU [1]) derived from table S3. Standardized food utility index values are from the literature [2]. Analyses were limited to high survivorship elements [3]: cranium, mandible, humerus, radius, metacarpal, femur, tibia, and metatarsal. Analyses were run separately for small and medium-sized bovids, and for conditions with and without the tongue.

1. Lyman RL (1994) Vertebrate Taphonomy. Cambridge: Cambridge University Press.

2. Metcalfe D, Jones KT (1988) A reconsideration of animal body part utility indices. Am Antiq 53: 486-504.

3. Marean CW, Cleghorn N (2003) Large mammal skeletal element transport: applying foraging theory in a complex taphonomic system. Journal of Taphonomy 1: 15-42.
