# Supplementary material for: Earliest Archaeological Evidence of Persistent Hominin Carnivory
Source: PLoS One. 2013 Apr 25;8(4):e62174. doi: 10.1371/journal.pone.0062174 (PMC3636145; doi:10.1371/journal.pone.0062174)
Supplement: Table S6 — Skeletal element abundances and within-bone nutrients. (DOC) [file pone.0062174.s006.doc]

**Table S6. Skeletal element abundances and within-bone nutrients.**

| **Bed** | **Body size** | **N** | **Spearman's rho** | **Significance (2-tailed)** |
| --- | --- | --- | --- | --- |
| KS-1 | Small | 6 | 0.265 | 0.612 |
|  | Medium | 6 | 0.657 | 0.156 |
| KS-2 | Small | 6 | -0.618 | 0.191 |
|  | Medium | 6 | 0.580 | 0.228 |
| KS-3 | Small | 6 | -0.507 | 0.305 |
|  | Medium | 6 | 0.407 | 0.423 |

**Table S6.** Correlation coefficients (r_s_) between skeletal element abundances and within-bone nutrients. Skeletal element abundance data (minimum animal units; MAU [1]) derived from table S3. Within-bone nutrient data are wet marrow weights standardized to the tibia. We use ‘mean size 1&2 adults’ and ‘wildebeest’ data for small and medium-sized bovid analyses, respectively [2]. Analyses were limited to high survivorship elements [3]: humerus, radius, metacarpal, femur, tibia, and metatarsal.

1. Lyman RL (1994) Vertebrate Taphonomy. Cambridge: Cambridge University Press.

2. Blumenschine RJ, Madrigal TC (1993) Variability in long bone marrow yields of East African ungulates and its zooarchaeological implications. J Archaeol Sci 20: 555-587.

3. Marean CW, Cleghorn N (2003) Large mammal skeletal element transport: applying foraging theory in a complex taphonomic system. Journal of Taphonomy 1: 15-42.
