# Supplementary material for: Earliest Archaeological Evidence of Persistent Hominin Carnivory
Source: PLoS One. 2013 Apr 25;8(4):e62174. doi: 10.1371/journal.pone.0062174 (PMC3636145; doi:10.1371/journal.pone.0062174)
Supplement: Table S7 — Skeletal element abundances and within-bone resource extraction rates. (DOC) [file pone.0062174.s007.doc]

**Table S7. Skeletal element abundances and within-bone resource extraction rates.**

| **Bed** | **Body size** | **Condition** | **N** | **Spearman's rho** | **Significance (2-tailed)** |
| --- | --- | --- | --- | --- | --- |
| KS-1 | Small | High fat | 7 | 0.436 | 0.328 |
|  |  | Moderate fat |  | 0.473 | 0.284 |
|  |  | Low fat |  | 0.055 | 0.908 |
|  |  | Ext. low fat |  | 0.505 | 0.248 |
| KS-1 | Medium | High fat | 7 | 0.327 | 0.474 |
|  |  | Moderate fat |  | 0.358 | 0.431 |
|  |  | Low fat |  | 0.582 | 0.170 |
|  |  | Ext. low fat |  | 0.655 | 0.111 |
| KS-2 | Small | High fat | 7 | -0.327 | 0.474 |
|  |  | Moderate fat |  | -0.145 | 0.756 |
|  |  | Low fat |  | -0.055 | 0.908 |
|  |  | Ext. low fat |  | 0.073 | 0.876 |
| KS-2 | Medium | High fat | 7 | 0.107 | 0.819 |
|  |  | Moderate fat |  | 0.162 | 0.728 |
|  |  | Low fat |  | 0.536 | 0.215 |
|  |  | Ext. low fat |  | 0.500 | 0.253 |
| KS-3 | Small | High fat | 7 | -0.059 | 0.900 |
|  |  | Moderate fat |  | 0.177 | 0.704 |
|  |  | Low fat |  | 0.473 | 0.284 |
|  |  | Ext. low fat |  | 0.268 | 0.561 |
| KS-3 | Medium | High fat | 7 | 0.225 | 0.628 |
|  |  | Moderate fat |  | 0.274 | 0.552 |
|  |  | Low fat |  | 0.524 | 0.227 |
|  |  | Ext. low fat |  | 0.580 | 0.172 |

**Table S7.** Correlation coefficients (r_s_) between skeletal element abundances and within-bone resource extraction rates. Skeletal element abundance data (minimum animal units; MAU [1]) derived from table S3. Return rates follow the literature for ‘high fat’, ‘moderate fat’, ‘low fat’, and ‘extremely low fat’ animal conditions [2]. We use ‘young adult male impala’ and ‘adult female wildebeest’ data for small and medium-sized bovid analyses, respectively [2]. Analyses were limited to high survivorship elements [3,4]: ‘head’ (either the cranium or mandible MAU value, whichever is greater), humerus, radius, metacarpal, femur, tibia, and metatarsal.

1. Lyman RL (1994) Vertebrate Taphonomy. Cambridge: Cambridge University Press.

2. Lupo KD (1998) Experimentally derived extraction rates for marrow: implications for body part exploitation strategies of Plio-Pleistocene hominid scavengers. J Archaeol Sci 25: 657-675.

3. Marean CW, Cleghorn N (2003) Large mammal skeletal element transport: applying foraging theory in a complex taphonomic system. Journal of Taphonomy 1: 15-42.

4. Madrigal TC, Blumenschine RJ (2000) Preferential processing of high return rate marrow bones by Oldowan hominids: a comment on Lupo. J Archaeol Sci 27: 739-741.
