# Supplementary material for: Earliest Archaeological Evidence of Persistent Hominin Carnivory
Source: PLoS One. 2013 Apr 25;8(4):e62174. doi: 10.1371/journal.pone.0062174 (PMC3636145; doi:10.1371/journal.pone.0062174)
Supplement: Table S8 — Skeletal element evenness. Evenness calculated using the Shannon evenness index. Bone frequencies modeled using a Bayesian multinomial model. (DOC) [file pone.0062174.s008.doc]

**Table S8.** **Skeletal element evenness**.

| **Bed** | **Body Size** | **N** | **Mean** | **SD** | **95% Credible Interval** |
| --- | --- | --- | --- | --- | --- |
| KS-1 | Small | 8 | 0.9524 | 0.0202 | 0.9071 to 0.9848 |
|  | Medium | 8 | 0.9192 | 0.0281 | 0.8567 to 0.9666 |
| KS-2 | Small | 8 | 0.9550 | 0.0184 | 0.9134 to 0.9849 |
|  | Medium | 8 | 0.9444 | 0.0183 | 0.9043 to 0.9758 |
| KS-3 | Small | 8 | 0.9239 | 0.0340 | 0.8455 to 0.9771 |
|  | Medium | 8 | 0.8076 | 0.0575 | 0.6858 to 0.9091 |

**Table S8.** Evenness was calculated using the Shannon evenness index [1-2]. Skeletal element abundance data (minimum animal units; MAU [3]) were derived from table S3, and limited to ‘high survival elements’ [4]: cranium, mandible, humerus, radius, metacarpal, femur, tibia, and metatarsal. Within each assemblage and animal size group (small vs. medium), bone frequencies were modeled with a Bayesian multinomial model using a non-informative Dirichlet prior on the probabilities for each bone type [5]. This was equivalent to placing uniform priors on each probability. Markov chain Monte Carlo methods were used to fit the model, as implemented in the statistical package WinBugs. Two parallel chains of 5000 were used, after a burn-in of 1000 iterations. Initial values for the chains were randomly generated. Convergence was checked using the Gelman-Rubin statistic. The posterior distributions for each bone type were then combined to form a posterior distribution for the Shannon evenness index. The index is a measure of how evenly distributed items are across categories. An evenness value of one indicates a perfectly even distribution. Smaller values indicate less even distribution. The table summarizes the posteriors for each assemblage by animal size group. None of the 95% credible intervals contains 1, thus no assemblage has a perfectly even distribution [see also 1,5-7].

1. Faith JT, Gordon AD (2007) Skeletal element abundances in archaeofaunal assemblages: economic utility, sample size, and assessment of carcass transport strategies. J Archaeol Sci 34: 872-882.

2. Magurran AE (1988) Ecological Diversity and its Measurement. Princeton: Princeton University Press.

3. Lyman RL (1994) Vertebrate Taphonomy. Cambridge: Cambridge University Press.

4. Marean CW, Cleghorn N (2003) Large mammal skeletal element transport: applying foraging theory in a complex taphonomic system. Journal of Taphonomy 1: 15-42.

5. Seaman JW III (2010) Topics in Bayesian inference: induced priors, proof loading for combination drugs, and distribution of archaeological skeletal assemblages [PhD dissertation]. Waco, Texas: Baylor University.

6. Egeland CP, Dominguez-Rodrigo M (2008) Taphonomic perspectives on hominid site use and foraging strategies during Bed II times at Olduvai Gorge, Tanzania. J Hum Evol 55: 1031-1052.

7. Faith JT, Dominguez-Rodrigo M, Gordon AD (2009) Long-distance carcass transport at Olduvai Gorge? A quantitative examination of Bed I skeletal element abundances. J Hum Evol 56: 247-256.
